# The influence of probable rapid eye movement sleep behavior disorder and sleep insufficiency on fall risk in a community-dwelling elderly population

**DOI:** 10.1186/s12877-021-02513-2

**Published:** 2021-10-27

**Authors:** Chao Han, Jing An, Piu Chan

**Affiliations:** 1 National Clinical Research Center for Geriatric Disorders, Xuanwu Hospital of Capital Medical University, Beijing, China; 2 Department of Neurobiology, Neurology and Geriatrics, Clinical Center for Parkinson’s Disease, Key Laboratories for Neurodegenerative Diseases of the Ministry of Education, Beijing Key Laboratory for Parkinson’s Disease, Advanced Innovative Center for Human Brain Protection, Beijing Institute of Geriatrics, Parkinson Disease Center of Beijing Institute for Brain Disorders, Xuanwu Hospital of Capital Medical University, 45 Changchun Road, Beijing, 100053 China

**Keywords:** Probable rapid eye movement sleep behavior disorder, Sleep insufficiency, Fall, Interaction, Elderly

## Abstract

**Background:**

The objective was to investigate the individual effect and potential interactions of probable rapid eye movement sleep behavior disorder (pRBD) and sleep insufficiency on fall risk among a Chinese elderly population.

**Methods:**

Community-dwelling population aged 55 years or above were recruited from the Beijing Longitudinal Study on Aging II cohort from 2010 to 2011. Odds ratio (ORs) and 95% confidence intervals (CIs) were estimated using multivariate logistic regression models. Multiplicative and additive interactions between pRBD and sleep insufficiency were examined using likelihood ratio tests and relative excess risk due to interaction (RERI), respectively.

**Results:**

Among 6891 included participants, 479 experienced at least once fall. pRBD and sleep insufficiency were both independently associated with elevated fall risk. Compared to the elderly without pRBD or sleep insufficiency, pRBD and sleep insufficiency was each associated with a 2.57-fold (OR = 2.57, 95%CI: 1.46–4.31) and 1.45-fold (OR = 1.45, 95%CI: 1.11–1.88) risk of falls individually, while their coexistence was associated with a less-than-additive 17% (OR = 1.17, 95%CI: 0.43–2.63) increased risk of falls. The combination of these two factors demonstrated evidence of a negative interaction on both multiplicative (ratio of ORs = 0.31, 95%CI: 0.10, 0.86) and additive (RERI = − 1.85, 95%CI: − 3.61, − 0.09) scale.

**Conclusions:**

Our study has provided robust evidence for the adverse effect of pRBD and sleep insufficiency, as well as their negative interaction on increasing fall risk in a Chinese elderly population.

**Supplementary Information:**

The online version contains supplementary material available at 10.1186/s12877-021-02513-2.

## Background

Falls, as a common event and major health problem for the elderly, has been identified as one of the leading causes of injury, disability, hospitalization, and mortality in people aged 60 years or older [1]. It was reported that at least one-third of elderly population experienced falls once or more per year [2]. Increasing epidemiological studies have identified multiple risk factors associated with falls, including physical disability, cognitive impairment, diabetes, medication use, etc. [3] However, these studies are far from enough with many critical factors not yet been thoroughly investigated.

Rapid eye movement sleep behavior disorder (RBD) is a parasomnia characterized by vivid dreams, complex dream-enacting behaviors, and loss of normal muscle atonia during rapid eye movement (REM) sleep [4]. Idiopathic RBD has been well-recognized as a risk factor predisposing to various neurodegenerative synucleinopathies including Parkinson’s disease (PD), dementia with Lewy bodies and multiple system atrophy [5, 6]. In recent years, studies gradually shifted their attention to the potential relationship between RBD and motor manifestations especially falls among PD patients and yielded conflicting results [7–14]. Nonetheless, all these studies were conducted among PD patients while general population-based evidence was quite scarce regarding falls. Besides, the relationship of sleep duration with fall risk also remains contested. Despite an abundance of studies showing that sleep insufficiency was associated with increased fall risk among both elder and younger adults [15–18], others have reported an elevated risk of falls among elders with excessive nocturnal or daily sleep [19, 20].

Given that RBD and sleep insufficiency represent sleep quality and quantity problems respectively and join together to mediate effects on brain function [21], it is reasonable to infer a possible interplay between them on influencing fall risk. This interplay might partially explain the inconsistency of existing evidence regarding the relationship of RBD and sleep duration with falls occurrence. Besides, understanding how they interact with each other might provide an important implication for developing plausible interventions in preventing falls among the growing elderly population. To our knowledge, there is no published study ever exploring this relationship as yet.

To fill this knowledge gap, we conducted a large community-based study to investigate the potential impact of probable RBD (pRBD) and sleep insufficiency on fall risk and examine the presence of interactions between these two sleep disorders among a Chinese elderly population.

## Materials and Methods

### Study participants

**Fig. 1:**
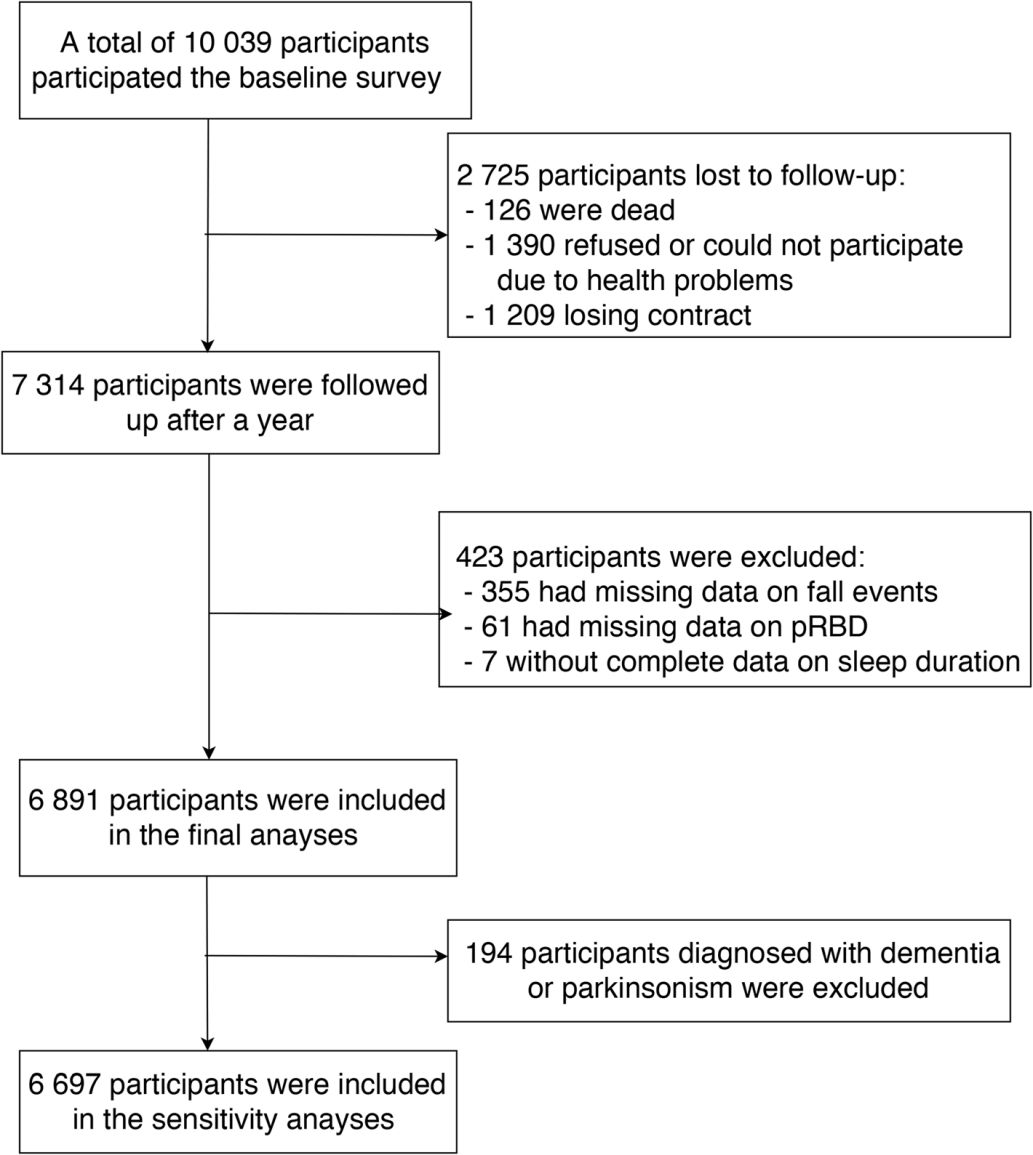
Flowchart of study recruitment

The study participants were enrolled from the Beijing Longitudinal Study on Aging II (BLSA-II), a large community-based prospective cohort study. Detailed information of this study has been published previously [22]. Briefly, a multistage cluster random sampling method was used to select a representative community-dwelling population aged 55 years and older from three urban districts and one rural district in Beijing, China. A total of 10,039 participants were recruited at baseline from July to November in 2009, among which 7314 continued participating the first follow-up from August 2010 to January 2011. Our study was based on data obtained during the follow up period. We excluded 355 participants due to incomplete information on fall events, 61 with missing data on pRBD, and 7 without sleep duration information, leaving 6891 subjects involved in the final analyses. Figure 1 showed the flowchart of this study. Nearly no significant difference was detected between included and excluded participants (**Supplementary Table** **1**). The study protocol was approved by the Research Ethics Committee of Xuanwu Hospital of Capital Medical University. All participants had provided informed consent.

### Definition of falls, pRBD, and sleep insufficiency

A fall was defined as an unintentional coming to rest on the ground or lower level, with or without loss of consciousness [23]. Subjects were classified as fallers according to a numerical answer greater than zero to the question “During the preceding 12 months, how many times have you unintentionally lost your balance and land on the ground or lower level?”. The presence of pRBD was determined with a validated RBD questionnaire-Hong Kong (RBD-HK). RBD-HK was composed of 13 items regarding the presence, frequency, and severity of RBD symptoms, and proved to have a good sensitivity (82.2%) and specificity (86.9%) in screening RBD among general population [24]. An RBD-HK score of 19 or more can be diagnosed as pRBD, as video-polysomnography is required to objective establish the diagnosis [25]. Sleep duration was measured as self-reported average hours of sleep per night during the past year and categorized into ≥6 and < 6 h for analysis while the latter group was deemed as sleep insufficiency.

### Data collection

A standardized structured questionnaire was administered to collect information on sociodemographic characteristics, lifestyle factors, medical histories, and comorbidities by trained investigators in a face-to-face interview. Basic sociodemographic characteristics included age, sex, education level, occupation, marital status, living type, and average household income. Educational level was classified into three categories: illiteracy or primary school, middle or high school, and university or higher. Occupation was categorized as unemployed, worker or farmer, professional technician or others. Marital status was dichotomized into married or partnered and never married or non-partnered. Living type was categorized as living alone or living with others. Average monthly household income was divided into < 3000 yuan and ≥ 3000 yuan. Self-reported smoking and drinking status was uniformly categorized as never or former consumers and current consumers. Physical activity was assessed by average daily hours they spent on doing exercise (≤/> 30 min). Two important dietary habits were also evaluated by frequency including protein intake (high intake: more than two high-protein dietary habits involving at least one daily serving of dairy; two or more weekly servings of soy products/eggs; and daily intake of meat/fish/poultry) and consumption of fruits and vegetables (high intake: more than twice per day). Height and weight were measured with participants wearing light clothes and bare foot. Body mass index (BMI) was calculated by dividing weight in kilograms by height in meters squared, with a threshold of overweight or obesity setting at 24 kg/m^2^. Medical histories of common chronic diseases including stroke, coronary heart disease (CHD), hypertension, diabetes, hyperlipidemia, and hyperuricemia were confirmed based on a combination of self-reported physician’s diagnosis, treatment history, and clinical examinations. Parkinson’s disease was screened using a validated Parkinson screening questionnaire. Self-reported history of a clinical diagnosis of dementia and parkinsonism, fall history, and family history of parkinsonism and dementia were also inquired. The presence of hunchback was assessed with the question “Have you noticed a recent decrease in height or hunchback?”. Visual impairment was diagnosed according to an affirmative answer to the question “Have you experienced visual problems recently (i.e., having a corrected vision below 0.3)?” [26, 27]. Fear of falling was also appraised by asking the participants if they were fearful or worried about falling in recent months.

Geriatric clinical assessments included cognitive function (Mini-Mental State Examination, MMSE), depressive status (15-item Geriatric Depression Scale, GDS-15), functional capacity (Activities of Daily Living, ADL; Instrumental Activities of Daily Living, IADL), and gait and balance performance (Tinetti Mobility Test, TMT). Subjects with MMSE score < 24 implied having cognitive impairment. GDS score > 7 indicated depressive mood. Barthel index was calculated to evaluate basic functional capacity according to ADL score while subjects with a Barthel index < 100 were considered to have basic dysfunction. The IADL score was obtained through evaluating 8 instrumental activities and yielded a final score ranging from 8 to 32. For the convenience of analysis, the IADL score was divided into three groups at equal intervals (8–16/16–24/24–32) while the latter two groups indicated higher instrumental dysfunction. The maximum total score of TMT was 28 while subjects who scored < 15 were defined as having gait and balance impairment.

### Statistical analyses

Descriptive statistics were used to summarize the characteristics of participants by subgroups of fallers and non-fallers. Continuous variables were described as mean ± standard deviation (SD) and compared by t test, while categorical variables were described as percentages and compared by Chi-square test. Logistic regression models were used to evaluate the association of pRBD status and sleep duration with fall risk by calculating odds ratios (ORs) and corresponding 95% confidence intervals (CIs). The minimal model controlled for age and sex. The multivariate model controlled for age, sex, education level, marital status, occupation, residence type, family income, smoking status, drinking status, physical activity, protein intake, fruits and vegetables intake, BMI, family history of parkinsonism or dementia, fall history, and various clinical comorbidities (stroke, CHD, hypertension, diabetes, hyperlipidemia, hyperuricemia, visual impairment, hunchback, cognitive impairment, depression, basic and instrumental dysfunction). The full model additionally controlled for fear of falling and gait and balance impairment, two well-established risk factors of falls [3].

We tested for the presence of both multiplicative and additive interactions between pRBD and sleep insufficiency on fall risk simultaneously. Potential multiplicative interaction was examined by including a cross-product term in the full logistic model and compared with the prior model using likelihood ratio test. The relative excess risk due to interaction (RERI) was calculated to estimate additive interaction [28]. RERI refers to the excess risk attributed to interaction relative to the risk without exposure and is supposed to equal 0 in the absence of additive interaction. A statistically significant RERI > 0 indicates positive biological interaction in which the combined effect of two factors is greater than the sum of individual effect of each factor. A statistically significant RERI < 0 implies negative biological interaction in which the joint effect is less than the sum of individual effects. The δ method from Hosmer and Lemeshow was used to calculate variances of RERI estimates [29]. To further illustrate potential modification effect between these factors, we conducted two separate analyses stratified by pRBD status and sleep duration, respectively. Finally, we performed sensitivity analyses in a restricted sample after excluding those with a self-reported diagnosis of dementia or parkinsonism (*n* = 194). Two-tailed *P* values < 0.05 were considered statistically significant. All statistical analyses were performed using the R software (version 3.5.1; R Development Core Team 2018, www.R-project.org).

## Results

The characteristics of study participants are shown in Table 1, overall and by falling status. The mean age of included participants was 71.4 years, and 61.4% were female. A total of 479 (7.0%) participants once fell during the past year. Risk of falls didn’t vary significantly by education level, occupation, average income, smoking or drinking status, physical activity, protein intake, family history of parkinsonism or dementia, and certain medical conditions including overweight or obesity, hypertension, and diabetes. Whereas, falls were more common in female, older adults, those who were never married or non-partnered, and living alone. Besides, fallers tended to take fruits and vegetables more frequently, sleep less, bear greater burden of chronic comorbidities, and display worse cognitive, psychological, and physical performance than non-fallers. We also observed a considerably higher prevalence of fear of falling and gait and balance impairment among fallers.

**Table 1:** Demographic and clinical characteristics of the study participants

	Overall (*n* = 6891)	Fallers (*n* = 479)	Non-fallers (*n* = 6412)	*P* Value
Age, y	71.4 ± 7.4	73.7 ± 7.1	71.2 ± 7.4	< 0.001
Age group				
55–64 years	1422 (20.6)	56 (11.7)	1366 (21.3)	< 0.001
65–74 years	2970 (43.1)	199 (41.5)	2771 (43.2)	
75–79 years	1571 (22.8)	133 (27.8)	1438 (22.4)	
> =80 years	928 (13.5)	91 (19.0)	837 (13.1)	
Sex				
Male	2732 (39.6)	152 (31.7)	2580 (40.2)	< 0.001
Female	4159 (60.4)	327 (68.3)	3832 (59.8)	
Education level				
Primary school or lower	2560 (37.5)	195 (41.1)	2365 (37.3)	0.224
Middle or high school	3343 (49.0)	216 (45.5)	3127 (49.3)	
University or higher	915 (13.4)	64 (13.5)	851 (13.4)	
Occupation				
Unemployed	2237 (32.8)	167 (35.2)	2070 (32.6)	0.088
Worker or farmer	3355 (49.2)	211 (44.4)	3144 (49.5)	
Professional technician or others	1229 (18.0)	97 (20.4)	1132 (17.8)	
Marital status				
Married or partnered	5590 (81.2)	351 (73.3)	5239 (81.7)	< 0.001
Never married or non-partnered	1298 (18.8)	128 (26.7)	1170 (18.3)	
Residence type				
Living with others	6389 (92.8)	429 (89.6)	5960 (93.0)	0.007
Living alone	499 (7.2)	50 (10.4)	449 (7.0)	
Average monthly household income				
< 3000 yuan	5176 (80.2)	349 (78.1)	4827 (80.3)	0.275
> =3000 yuan	1280 (19.8)	98 (21.9)	1182 (19.7)	
Smoking status				
Never or former smoking	6126 (88.9)	426 (88.9)	5700 (88.9)	0.999
Current smoking	765 (11.1)	53 (11.1)	712 (11.1)	
Drinking status				
Never or former smoking	6013 (87.3)	423 (88.3)	5590 (87.2)	0.520
Current drinking	878 (12.7)	56 (11.7)	822 (12.8)	
Physical activity				
< = 30 min/day	1662 (24.1)	127 (26.5)	1535 (23.9)	0.224
> 30 min /day	5229 (75.9)	352 (73.5)	4877 (76.1)	
Protein intake				
Low	1979 (28.7)	141 (29.4)	1838 (28.7)	0.758
High	4912 (71.3)	338 (70.6)	4574 (71.3)	
Fruits and vegetables intake				
Low	1025 (14.9)	49 (10.2)	976 (15.2)	0.004
High	5866 (85.1)	430 (89.8)	5436 (84.8)	
Sleeping habits				
> = 6 h	5720 (83.0)	349 (72.9)	5371 (83.8)	< 0.001
< 6 h	1171 (17.0)	130 (27.1)	1041 (16.2)	
Family history of parkinsonism or dementia	50 (0.7)	5 (1.1)	45 (0.7)	0.560
Fear of falling	1896 (27.5)	271 (56.6)	1625 (25.3)	< 0.001
Fall history	442 (6.5)	80 (16.9)	362 (5.7)	< 0.001
Overweight or obese	4144 (60.1)	291 (60.8)	3853 (60.1)	0.813
Stroke	962 (14.0)	104 (21.7)	858 (13.4)	< 0.001
CHD	2019 (29.3)	175 (36.5)	1844 (28.8)	< 0.001
Hypertension	4613 (66.9)	325 (67.8)	4288 (66.9)	0.699
Diabetes	2755 (40.0)	211 (44.1)	2544 (39.7)	0.066
Hyperlipidemia	3940 (57.2)	300 (62.6)	3640 (56.8)	0.014
Hyperuricemia	1476 (21.4)	126 (26.3)	1350 (21.1)	0.008
Visual impairment	67 (1.0)	11 (2.4)	56 (0.9)	0.005
Hunchback	2232 (33.0)	258 (54.8)	1974 (31.3)	< 0.001
pRBD	210 (3.0)	31 (6.5)	179 (2.8)	< 0.001
Cognitive impairment	669 (9.8)	90 (19.0)	579 (9.1)	< 0.001
Depression	371 (5.4)	56 (11.9)	315 (5.0)	< 0.001
ADL score				
Barthel index = 100	940 (13.9)	136 (29.0)	804 (12.8)	< 0.001
Barthel index < 100	5808 (86.1)	333 (71.0)	5475 (87.2)	
IADL score				
8–16	6447 (95.5)	405 (86.4)	6042 (96.2)	< 0.001
16–24	192 (2.8)	39 (8.3)	153 (2.4)	
24–32	109 (1.6)	25 (5.3)	84 (1.3)	
Gait and balance impairment	270 (3.9)	47 (10.0)	223 (3.5)	< 0.001

*Abbreviations*: *CHD* coronary heart disease, *pRBD* probable rapid eye movement sleep behavior disorder, *ADL* activities of daily living, *IADL* instrumental activities of daily living

The presence of pRBD and/or sleep insufficiency were consistently associated with increased fall risk among elderly adults in both age- and sex-adjusted and multivariate regression models (Table 2). These associations were mildly attenuated but remained consistent even after adjusting for fear of falling and gait and balance impairment. It was evident that participants with insufficient sleep were at 34% (OR = 1.34, 95%CI: 1.03–1.73) higher risk of experiencing falls compared with their counterparts. Likewise, pRBD was independently associated with 75% (OR = 1.75, 95%CI: 1.08–2.74) increased risk of falls. However, their combined effect generated a less pronounced increase in fall risk compared with exposure to these factors individually: corresponding OR with 95%CI was 1.17 (0.43–2.63) vs. 2.57 (1.46–4.31) and 1.45 (1.11–1.88), respectively (Table 3). In stratified analyses, the deleterious effect of pRBD on fall risk was observed among participants with sufficient sleep but not among those lack of sleep. Analogously, sleep insufficiency was significantly associated with increased fall risk in the strata of non-pRBD participants but not among pRBD ones. These results implied that sleep insufficiency might negatively modify the impact of pRBD on fall risk and vice versa. In addition, we have observed a significant additive and multiplicative interaction between pRBD and sleep duration. The RERI was − 1.85 (95%CI: − 3.61, − 0.09, *P* = 0.019), meaning that the joint effect of pRBD and sleep insufficiency together was lower than expected from the sum of individual effects, namely there was a negative interaction on additive scale. Measure of interaction on multiplicative scale, the ratio of ORs, was 0.31 (95%CI: 0.10, 0.86, *P* = 0.031), meaning that the joint effect of pRBD and sleep insufficiency together was lower than expected from the product of individual effects, namely there was a negative interaction on multiplicative scale as well. In brief, regardless of whether assuming an additive or multiplicative scale, these two factors might interact antagonistically in relation to risk of falls. In sensitivity analyses where we excluded participants with dementia or parkinsonism, results remained nearly the same (**Supplementary Tables** **2** and 3).

**Table 2:** Association between probable rapid eye movement sleep behavior disorder (pRBD) and sleep insufficiency with risk of fall among a large community-dwelling elderly population

Characteristics	Age- and sex-adjusted logistic regression model		Multivariate logistic regression model (Model1^a^)		Full logistic regression model (Model2^b^)	
	OR (95%CI)	*P* value	OR (95%CI)	*P* value	OR (95%CI)	*P* value
pRBD status						
Non-pRBD	1.00 (reference)		1.00 (reference)		1.00 (reference)	
pRBD	2.57 (1.70–3.77)	< 0.001	1.98 (1.22–3.08)	0.004	1.75 (1.08–2.74)	0.018
Sleep duration						
> =6 h	1.00 (reference)		1.00 (reference)		1.00 (reference)	
< 6 h	1.7 (1.37–2.11)	< 0.001	1.32 (1.02–1.7)	0.031	1.34 (1.03–1.73)	0.028

^a^Model1 adjusted for age, sex, education level, marital status, occupation, residence type, family income, smoking status, drinking status, physical activity, protein intake, fruits and vegetables intake, BMI, family history of parkinsonism or dementia, fall history, and various clinical comorbidities (stroke, CHD, hypertension, diabetes, hyperlipidemia, hyperuricemia, visual impairment, hunchback, cognitive impairment, depression, ADL score, IADL score)

^b^Model2 adjusted all the above covariates plus fear of falling and gait and balance impairment

*Abbreviations*: *pRBD* probable rapid eye movement sleep behavior disorder, *OR* odds ratio, *CI* confidence interval, *BMI* body mass index, *CHD* coronary heart disease, *ADL* activities of daily living, *IADL* instrumental activities of daily living

**Table 3:** Interaction between probable rapid eye movement sleep behavior disorder (pRBD) and sleep insufficiency on the risk of fall among a large community-dwelling elderly population

	Non-pRBD		pRBD		OR (95% CI) for pRBD within strata of sleep duration
	N cases/controls	OR (95% CI)	N cases/controls	OR (95% CI)	
Sleep duration > = 6 h	328/5252	1.0 (reference)	21/119	2.57 (1.46–4.31) *P* = 0.001	2.57 (1.46–4.31) *P* = 0.001
Sleep duration < 6 h	120/981	1.45 (1.11–1.88) *P* = 0.006	10/60	1.17 (0.43–2.63) *P* = 0.735	0.81 (0.30–1.85) *P* = 0.638
OR (95% CI) for sleep duration within strata of pRBD status		1.45 (1.11–1.88) *P* = 0.006		0.45 (0.15–1.21) *P* = 0.130	

Measure of interaction on additive scale: RERI (95%CI) = −1.85 (−3.61, −0.09); *P* = 0.019

Measure of interaction on multiplicative scale: ratio of ORs (95%CI) = 0.31 (0.10, 0.86); *P* = 0.031

ORs are adjusted for age, sex, education level, marital status, occupation, residence type, family income, smoking status, drinking status, physical activity, protein intake, fruits and vegetables intake, BMI, family history of parkinsonism or dementia, fear of falling, fall history, and various clinical comorbidities (stroke, CHD, hypertension, diabetes, hyperlipidemia, hyperuricemia, visual impairment, hunchback, cognitive impairment, depression, ADL score, IADL score, gait and balance impairment)

*Abbreviations*: *pRBD* probable rapid eye movement sleep behavior disorder, *OR* odds ratio, *CI* confidence interval, *RERI* the relative excess risk due to interaction, *BMI* body mass index, *CHD* coronary heart disease, *ADL* activities of daily living, *IADL* instrumental activities of daily living

## Discussion

In this large-scale community-based study, we provided robust evidence that pRBD and sleep insufficiency were both associated with elevated fall risk among the general elderly population. Moreover, our study for the first time proposed that pRBD could interact antagonistically with sleep insufficiency in both additive and multiplicative scale to influence fall risk. We also raised the possibility that the adverse effect of pRBD on fall risk might be dependent on the presence of sufficient sleep, while sleep insufficiency was more likely to increase hazards of falls among non-pRBD patients.

Inconsistencies have been noted regarding the association of RBD and sleep duration with falls among the elderly based on existing evidence. In line with our findings, three cross-sectional studies reported a robust association between RBD and increased frequency of falls among idiopathic PD patients [7, 11, 12]. A most recent longitudinal study conducted in Italy also provided similar evidence that RBD could be deemed as an independent predictor of falls in PD patients [13]. Whereas several studies with relatively limited sample size once yielded negative results toward the same direction of association [8, 10, 14]. With respect to sleep duration, many pivotal studies have proved that sleep insufficiency should be deemed as a potent factor involved in falls and subsequent injuries for both elders [15, 16] and youngsters [17, 18]. Two representative cohort studies conducted by Stone found a 1.79 and 1.52-fold risk of having two or more falls in the following year among American old men and women who slept less than 5 h per night, respectively [15, 16]. Nevertheless, a few cross-sectional studies ever observed a positive relationship between excessive sleep and increased fall risk as well [19, 20]. These discrepancies may be explained by the heterogeneity in study design, study population, exposure assessment, data quality, and especially the overlook of potential interactions between some key factors. Furthermore, previous studies based on video polysomnography (PSG)-diagnosed RBD were usually small-sized which might limit their power to discover the true associations and result to negative findings by chance. In this regard, our study has extended existing findings by confirming the adverse effect of pRBD and sleep insufficiency on fall risk in consideration of potential interactions among a much larger community-dwelling population.

Sleep of good quality and sufficient duration has been supposed to benefit brain function and health status [30]. Whereas pathophysiological mechanisms underlying the association of pRBD and sleep insufficiency with increased fall risk are yet undetermined. There are several potential explanations accounting for the relationship between pRBD and fall risk. First of all, RBD has been established as one of the earliest and most specific prodromal signs of progressive neurodegenerative diseases involving α-synuclein pathology, including PD, dementia with Lewy bodies, and multiple system atrophy [31]. The onset of RBD usually precedes the development of neurodegeneration by several years. According to a latest review by Galbiati, the average conversion rate from isolated/idiopathic RBD to an overt neurodegenerative syndrome was 33.5% at 5 years follow-up, 82.4% at 10.5 years and 96.6% at 14 years [31]. Besides, many biomarkers of neurodegeneration have been reported to be associated with isolated RBD, ranging from neurophysiology, cognitive decline, neuroimaging findings, motor dysfunction to autonomic impairment [32]. For instance, a wearable-based, real-world gait monitoring study has detected reduced gait velocity, variability, and rhythm in RBD patients compared with their age-matched controls [33]. Laboratory assessments also revealed deficits in postural control and footstep asymmetry during dual-task walking in idiopathic RBD patients compared with controls [34]. Notably, RBD is generally preceded by these motor deficits including posture instability and gait disorders which might increase the tendency to falls [35, 36]. The presence of more axial symptoms, freezing gait, and akinetic-rigid subtype in PD patients with RBD also verified this kind of speculation [37–39]. On the other hand, autonomic symptoms encompassing orthostatic hypotension (OH) is also common in RBD patients [32] while OH has been well-recognized to pose high risk of falls among older adults [40]. According to Pilotto’s systematic review, numerous studies suggested a closely linked diad of OH and RBD among PD patients while the OH-RBD cluster generally induced increased postural sway in standing and more severe impairment of static balance [41]. Alternatively, several etiological mechanisms are plausible. Neuropathologic case reports have demonstrated signs of α-synuclein deposition in the locus coeruleus [42, 43], thalamic and neocortical cholinergic deficits [44], and involvement of pedunculopontine nucleus in the locomotor mesencephalic area [45] in RBD patients, all of which are critical in modulating gait and postural stability. Likewise, a variety of mechanisms might mediate the association between sleep insufficiency and risk of falls. Insomnia, a common cause of sleep insufficiency, is also a precursor and major sleep complain of neurodegenerative diseases as the case with pRBD [46]. It can be speculated that many motor deficits are already present at the presence of insomnia. Besides, untreated insomnia and resulting sleep insufficiency may exacerbate cognitive and behavioral symptoms in subjects with synucleinopathies [47]. A meta-analysis consistently confirmed the negative effects of sleep insufficiency on mood, cognitive function, and motor performance among the general populations [48]. Sleep deprivation could also result in slower reaction time [49, 50], worse attention [51], memory deficits [49], and postural imbalance [17, 51]. The destabilizing effects of sleep insufficiency was particularly more pronounced among elders than youngsters [52]. Other etiological hypotheses included cerebral white matter lesions caused by early morning awaking [53] and poor muscle strength secondary to chronic inflammation triggered by sleep deprivation [54, 55]. All the above factors have been well acknowledged as contributors to falls [3, 56, 57].

Most intriguingly, our study has proposed that pRBD patients with sufficient sleep exhibited the highest risk of falls, followed by individual exposure to sleep deprivation and a combination thereof, implying a significant negative interaction between pRBD and sleep insufficiency. Interpretation of this finding is complex. One possible explanation is that long sleep duration, especially long REM sleep, might increase the episode and frequency of pRBD, consequently amplifying the detrimental effect of pRBD on falls. On the contrary, sleep insufficiency might lead to an attenuated effect of pRBD on fall risk. This could also explain the observed adverse effect of pRBD on falls exclusively among those with sufficient sleep. Another possible explanation is these two exposures might operate on sharing biological pathways whereby they compete to affect fall risk. Nevertheless, there is currently limited evidence on potential pathways. Further verification of our findings along with experimental studies probing into relevant biological pathways are warranted.

Our findings are of substantial public health significance and highlight the importance of clinical assessment and management of sleep-related problems among the general elderly population. Given the high prevalence of sleep disorders in the elderly [49], uniformly inquiring about sleep insufficiency and pRBD in clinical practice may be useful in identifying high-risk population who require sleep hygiene interventions to prevent falls. Moreover, it’s notably worthy to be mentioned that sleep insufficiency and pRBD were both significantly associated with another major public health problem among middle-aged and elder adults: cognitive decline and dementia [35, 47, 58, 59]. A most recent large-scale cohort study reported higher dementia risk associated with a sleep duration of 6 h or less at age 50 or 60 compared with a normal sleep duration [58]. Emerging research also provided a direct link between sleep disturbance with dementia pathophysiology, suggesting that treating sleep disorders may target basic mechanisms of cognitive decline [60]. It can be speculated that addressing sleep disturbance and reasonably prolonging sleep time would substantially reduce falls, dementia, and related injuries among the elderly.

Yet, these results should also be interpreted in light of some limitations. Firstly, the intrinsic essence of a cross-sectional study might hinder us to conclude a causal relationship between pRBD, sleep insufficiency, and falls. Although the large sample size, standardized data-collection protocols and stringent quality control procedures enabled us to identify the true association, more large-scale and well-designed longitudinal studies are warranted to validate our findings in the future. Secondly, the diagnosis of pRBD using a self-administered questionnaire might lead to misclassification bias and underestimation of the true association. Besides, all the sociodemographic and lifestyle information were collected via a retrospective questionnaire and probably subject to recall bias. The possibility of residual confounding by some unavailable risk factors can’t be ruled out either.

Notwithstanding these limitations, several strengths of our study deserve mention. To the best of our knowledge, this is the first attempt to investigate the potential interplay between pRBD and sleep insufficiency in relation to fall risk using a large community-based population. The major strength of our study is the large sample size, community-based study design, and accessibility to a wide variety of potential confounders. Furthermore, prior studies exploring the relationship between RBD and fall risk were mostly based on PSG-diagnosed cases among PD patients in clinical settings. These patients generally suffered a worse form of RBD and exhibited disparate characteristics compared with the general population. In this regard, our study has provided additional evidence based on questionnaire screened pRBD in the general population for the first time. Whereas more studies are warranted to investigate the suggestive associations and underlying pathogeneses. Considering subsequent health hazards caused by falls, future studies should further examine more detailed fall outcomes including disability, hospitalization, and medical health burden in association with subjective sleep quality. Notably, these associations should be investigated under consideration of potential modification by sleep duration.

## Conclusions

Overall, we have found a robust relationship of pRBD and sleep insufficiency with fall risk among the community-dwelling Chinese elderly population. Sleep insufficiency might negatively modify the detrimental effect caused by pRBD on falls. Further verification of our findings in prospective studies as well as investigations probing into underlying biological mechanisms are warranted in the future.

## Supplementary Information

**Additional file 1 Supplementary Table 1**. Baseline characteristics of included participants versus excluded participants.

**Additional file 2 Supplementary Table 2**. Association between probable rapid eye movement sleep behavior disorder (pRBD) and sleep insufficiency with risk of fall among participants without dementia or parkinsonism.

**Additional file 3 Supplementary Table 3**. Interaction between probable rapid eye movement sleep behavior disorder (pRBD) and sleep insufficiency on the risk of fall among participants without dementia or parkinsonism.

## Data Availability

The source data of BLSA-II project are not accessible to the public due to privacy regulations in China. Only team members who participated in this project are allowed to acquire the raw data.

## Abbreviations

pRBD: Probable rapid eye movement sleep behavior disorder
REM: Rapid eye movement
PD: Parkinson’s disease
BLSA-II: Beijing Longitudinal Study on Aging II
BMI: Body mass index
CHD: Coronary heart disease
MMSE: Mini-Mental State Examination
GDS-15: 15-item Geriatric Depression Scale
ADL: Activities of Daily Living
IADL: Instrumental Activities of Daily Living
TMT: Tinetti Mobility Test
SD: Standard deviation
ORs: Odds ratios
CIs: Confidence intervals
RERI: Relative excess risk due to interaction
PSG: Polysomnography
OH: Orthostatic hypotension
