# Supplementary material for: The influence of probable rapid eye movement sleep behavior disorder and sleep insufficiency on fall risk in a community-dwelling elderly population
Source: BMC Geriatr. 2021 Oct 27;21:606. doi: 10.1186/s12877-021-02513-2 (PMC8549138; doi:10.1186/s12877-021-02513-2)
Supplement: Supplementary file 1 — Additional file 1 Supplementary Table 1. Baseline characteristics of included participants versus excluded participants. [file 12877_2021_2513_MOESM1_ESM.docx]

**Supplementary Table1. Baseline characteristics of included participants versus excluded participants.**

|  | Overall  (n=7314) | Included  (n=6891) | Excluded  (n=423) | *P* Value |
| --- | --- | --- | --- | --- |
| Age, y | 71.4 ± 7.4 | 71.4 ± 7.4 | 71.1 ± 7.7 | 0.396 |
| Age group |  |  |  |  |
| 55-64 years | 1534 (21.0) | 1422 (20.6) | 112 (26.5) | 0.012 |
| 65-74 years | 3136 (42.9) | 2970 (43.1) | 166 (39.2) |  |
| 75-79 years | 1652 (22.6) | 1571 (22.8) | 81 (19.1) |  |
| >=80 years | 992 (13.6) | 928 (13.5) | 64 (15.1) |  |
| Sex |  |  |  |  |
| Male | 2902 (39.7) | 2732 (39.6) | 170 (40.2) | 0.865 |
| Female | 4412 (60.3) | 4159 (60.4) | 253 (59.8) |  |
| Education level |  |  |  |  |
| Primary school or lower | 2703 (37.4) | 2560 (37.5) | 143 (34.5) | 0.421 |
| Middle or high school | 3559 (49.2) | 3343 (49.0) | 216 (52.2) |  |
| University or higher | 970 (13.4) | 915 (13.4) | 55 (13.3) |  |
| Occupation |  |  |  |  |
| Unemployed | 2307 (31.9) | 2237 (32.8) | 70 (16.9) | <0.001 |
| Worker or farmer | 3595 (49.7) | 3355 (49.2) | 240 (57.8) |  |
| Professional technician or others | 1334 (18.4) | 1229 (18.0) | 105 (25.3) |  |
| Marital status |  |  |  |  |
| Married or partnered | 5930 (81.1) | 5590 (81.2) | 340 (81.0) | 0.969 |
| Never married or non-partnered | 1378 (18.9) | 1298 (18.8) | 80 (19.0) |  |
| Residence type |  |  |  |  |
| Living with others | 6773 (92.7) | 6389 (92.8) | 384 (91.4) | 0.359 |
| Living alone | 535 (7.3) | 499 (7.2) | 36 (8.6) |  |
| Average monthly household income |  |  |  |  |
| <3000 yuan | 5348 (80.1) | 5176 (80.2) | 172 (78.9) | 0.706 |
| >=3000 yuan | 1326 (19.9) | 1280 (19.8) | 46 (21.1) |  |
| Smoking status |  |  |  |  |
| Never or former smoking | 6478 (88.7) | 6126 (88.9) | 352 (85.6) | 0.052 |
| Current smoking | 824 (11.3) | 765 (11.1) | 59 (14.4) |  |
| Drinking status |  |  |  |  |
| Never or former smoking | 6359 (87.1) | 6013 (87.3) | 346 (84.2) | 0.084 |
| Current drinking | 943 (12.9) | 878 (12.7) | 65 (15.8) |  |
| Physical activity |  |  |  |  |
| <= 30 minutes/day | 1752 (24.0) | 1662 (24.1) | 90 (21.9) | 0.335 |
| > 30 minutes /day | 5550 (76.0) | 5229 (75.9) | 321 (78.1) |  |
| Protein intake |  |  |  |  |
| Low | 2070 (28.3) | 1979 (28.7) | 91 (21.5) | 0.002 |
| High | 5244 (71.7) | 4912 (71.3) | 332 (78.5) |  |
| Fruits and vegetables intake |  |  |  |  |
| Low | 1072 (14.7) | 1025 (14.9) | 47 (11.1) | 0.040 |
| High | 6242 (85.3) | 5866 (85.1) | 376 (88.9) |  |
| Sleeping habits |  |  |  |  |
| >= 6 hours | 6066 (83.1) | 5720 (83) | 346 (84.2) | 0.582 |
| < 6 hours | 1236 (16.9) | 1171 (17) | 65 (15.8) |  |
| Family history of parkinsonism or dementia | 55 (0.8) | 50 (0.7) | 5 (1.8) | 0.105 |
| Fear of falling | 1914 (27.6) | 1896 (27.5) | 18 (34.6) | 0.324 |
| Fall history | 468 (6.4) | 442 (6.5) | 26 (6.2) | 0.918 |
| Overweight or obese | 4390 (60.0) | 4144 (60.1) | 246 (58.2) | 0.450 |
| Stroke | 1018 (13.9) | 962 (14.0) | 56 (13.2) | 0.731 |
| CHD | 2102 (28.7) | 2019 (29.3) | 83 (19.6) | <0.001 |
| Hypertension | 4825 (66.0) | 4613 (66.9) | 212 (50.1) | <0.001 |
| Diabetes | 2935 (40.1) | 2755 (40.0) | 180 (42.6) | 0.319 |
| Hyperlipidemia | 4160 (56.9) | 3940 (57.2) | 220 (52.0) | 0.042 |
| Hyperuricemia | 1551 (21.2) | 1476 (21.4) | 75 (17.7) | 0.082 |
| Visual impairment | 70 (1.0) | 67 (1.0) | 3 (1.5) | 0.788 |
| Hunchback | 2285 (32.9) | 2232 (33.0) | 53 (30.3) | 0.507 |
| pRBD | 220 (3.1) | 210 (3.0) | 10 (6.8) | 0.019 |
| Cognitive impairment | 690 (9.8) | 669 (9.8) | 21 (10.6) | 0.799 |
| Depression | 381 (5.4) | 371 (5.4) | 10 (5.0) | 0.920 |
| ADL score |  |  |  |  |
| Barthel index = 100 | 978 (14.1) | 940 (13.9) | 38 (19.7) | 0.031 |
| Barthel index < 100 | 5963 (85.9) | 5808 (86.1) | 155 (80.3) |  |
| IADL score |  |  |  |  |
| 8-16 | 6633 (95.6) | 6447 (95.5) | 186 (96.4) | 0.799 |
| 16-24 | 197 (2.8) | 192 (2.8) | 5 (2.6) |  |
| 24-32 | 111 (1.6) | 109 (1.6) | 2 (1.0) |  |
| Gait and balance impairment | 278 (3.9) | 270 (3.9) | 8 (4.0) | 0.999 |

Abbreviations: CHD, coronary heart disease; pRBD, probable rapid eye movement sleep behavior disorder; ADL, activities of daily living; IADL, instrumental activities of daily living.
