# Supplementary material for: The influence of probable rapid eye movement sleep behavior disorder and sleep insufficiency on fall risk in a community-dwelling elderly population
Source: BMC Geriatr. 2021 Oct 27;21:606. doi: 10.1186/s12877-021-02513-2 (PMC8549138; doi:10.1186/s12877-021-02513-2)
Supplement: Supplementary file 2 — Additional file 2 Supplementary Table 2. Association between probable rapid eye movement sleep behavior disorder (pRBD) and sleep insufficiency with risk of fall among participants without dementia or parkinsonism. [file 12877_2021_2513_MOESM2_ESM.docx]

**Supplementary Table2. Association between probable rapid eye movement sleep behavior disorder (pRBD) and sleep insufficiency with risk of fall among participants without dementia or parkinsonism.**

|  | Age- and sex-adjusted  logistic regression model | |  | Multiple logistic  regression model (Model1^a^) | |  | Full logistic  regression model (Model2^b^) | |
| --- | --- | --- | --- | --- | --- | --- | --- | --- |
| Characteristics | OR (95%CI) | *P* value |  | OR (95%CI) | *P* value |  | OR (95%CI) | *P* value |
| pRBD status |  |  |  |  |  |  |  |  |
| Non-pRBD | 1.00 (reference) |  |  | 1.00 (reference) |  |  | 1.00 (reference) |  |
| pRBD | 2.53 (1.65-3.76) | <0.001 |  | 2.00 (1.21-3.16) | 0.005 |  | 1.78 (1.08-2.82) | 0.019 |
| Sleep duration |  |  |  |  |  |  |  |  |
| >=6 hours | 1.00 (reference) |  |  | 1.00 (reference) |  |  | 1.00 (reference) |  |
| < 6 hours | 1.67 (1.33-2.07) | <0.001 |  | 1.30 (1.01-1.69) | 0.048 |  | 1.32 (1.01-1.71) | 0.044 |

a. Model1 adjusted for age, sex, education level, marital status, occupation, residence type, family income, smoking status, drinking status, physical activity, protein intake, fruits and vegetables intake, BMI, family history of parkinsonism or dementia, fall history, and various clinical comorbidities (stroke, CHD, hypertension, diabetes, hyperlipidemia, hyperuricemia, visual impairment, hunchback, cognitive impairment, depression, ADL score, IADL score).

b. Model2 adjusted all the above covariates plus fear of falling and gait and balance impairment.

Abbreviations: pRBD, probable rapid eye movement sleep behavior disorder; OR, odds ratio; CI, confidence interval; BMI, body mass index; CHD, coronary heart disease; ADL, activities of daily living; IADL, instrumental activities of daily living.
