# Supplementary material for: The influence of probable rapid eye movement sleep behavior disorder and sleep insufficiency on fall risk in a community-dwelling elderly population
Source: BMC Geriatr. 2021 Oct 27;21:606. doi: 10.1186/s12877-021-02513-2 (PMC8549138; doi:10.1186/s12877-021-02513-2)
Supplement: Supplementary file 3 — Additional file 3 Supplementary Table 3. Interaction between probable rapid eye movement sleep behavior disorder (pRBD) and sleep insufficiency on the risk of fall among participants without dementia or parkinsonism. [file 12877_2021_2513_MOESM3_ESM.docx]

**Supplementary Table3. Interaction between probable rapid eye movement sleep behavior disorder (pRBD) and sleep insufficiency on the risk of fall among participants without dementia or parkinsonism.**

|  | Non-pRBD | |  | pRBD | | OR (95% CI) for pRBD  within strata of sleep duration |
| --- | --- | --- | --- | --- | --- | --- |
|  | N cases/controls | OR (95% CI) |  | N cases/controls | OR (95% CI) |  |
| Sleep duration >= 6 hours | 316/5117 | 1.0 (reference) |  | 19/114 | 2.70 (1.49-4.64)  *P* = 0.001 | 2.70 (1.49-4.64)  *P* = 0.001 |
| Sleep duration < 6 hours | 112/949 | 1.43 (1.08-1.87)  *P* = 0.011 |  | 10/60 | 1.18 (0.44-2.65)  *P* = 0.718 | 0.82 (0.30-1.89)  *P* = 0.674 |
| OR (95% CI) for sleep duration  within strata of pRBD status |  | 1.43 (1.08-1.87)  *P* = 0.011 |  |  | 0.44 (0.14-1.18) *P* = 0.116 |  |
| Measure of interaction on additive scale: RERI (95%CI) = -1.95 (-3.83, -0.08); P = 0.021.  Measure of interaction on multiplicative scale: ratio of ORs (95%CI) = 0.31 (0.10, 0.85); P = 0.029. | | | | | | |

ORs are adjusted for age, sex, education level, marital status, occupation, residence type, family income, smoking status, drinking status, physical activity, protein intake, fruits and vegetables intake, BMI, family history of parkinsonism or dementia, fear of falling, fall history, and various clinical comorbidities (stroke, CHD, hypertension, diabetes, hyperlipidemia, hyperuricemia, visual impairment, hunchback, cognitive impairment, depression, ADL score, IADL score, gait and balance impairment).

Abbreviations: pRBD, probable rapid eye movement sleep behavior disorder; OR, odds ratio; CI, confidence interval; RERI, the relative excess risk due to interaction; BMI, body mass index; CHD, coronary heart disease; ADL, activities of daily living; IADL, instrumental activities of daily living.
